# The RenTg Mice: A Powerful Tool to Study Renin-Dependent Chronic Kidney Disease

**DOI:** 10.1371/journal.pone.0052362

**Published:** 2012-12-26

**Authors:** Anne-Cecile Huby, Panagiotis Kavvadas, Carlo Alfieri, Ahmed Abed, Julie Toubas, Maria-Pia Rastaldi, Jean-Claude Dussaule, Christos Chatziantoniou, Christos E. Chadjichristos

**Affiliations:** 1 Institut National de la Santé et de la Recherche Médicale Joint Research Unit S 702, Batiment Recherche, Tenon Hospital, Paris, France; 2 Fondazione Istituto Di Ricovero e Cura a Carattere Scientifico, Ospedale Maggiore Policlinico & Fondazione D’Amico per la Ricerca sulle Malattie Renali, Milano, Italy; 3 Department of Physiology, AP-HP Hospital St Antoine, Paris, France; 4 Université Pierre et Marie Curie-Paris VI, Paris, France; Cedars-Sinai Medical Center, United States of America

## Abstract

**Background:**

Several studies have shown that activation of the renin-angiotensin system may lead to hypertension, a major risk factor for the development of chronic kidney disease (CKD). The existing hypertension-induced CDK mouse models are quite fast and consequently away from the human pathology. Thus, there is an urgent need for a mouse model that can be used to delineate the pathogenic process leading to progressive renal disease. The objective of this study was dual: to investigate whether mice overexpressing renin could mimic the kinetics and the physiopathological characteristics of hypertension-induced renal disease and to identify cellular and/or molecular events characterizing the different steps of the progression of CKD.

**Methodology/Principal Findings:**

We used a novel transgenic strain, the RenTg mice harboring a genetically clamped renin transgene. At 3 months, heterozygous mice are hypertensive and slightly albuminuric. The expression of adhesion markers such as vascular cell adhesion molecule-1 and platelet endothelial cell adhesion molecule-1 are increased in the renal vasculature indicating initiation of endothelial dysfunction. At 5 months, perivascular and periglomerular infiltrations of macrophages are observed. These early renal vascular events are followed at 8 months by leukocyte invasion, decreased expression of nephrin, increased expression of KIM-1, a typical protein of tubular cell stress, and of several pro-fibrotic agents of the TGFβ family. At 12 months, mice display characteristic structural alterations of hypertensive renal disease such as glomerular ischemia, glomerulo- and nephroangio-sclerosis, mesangial expansion and tubular dilation.

**Conclusions/Significance:**

The RenTg strain develops CKD progressively. In this model, endothelial dysfunction is an early event preceding the structural and fibrotic alterations which ultimately lead to the development of CKD. This model can provide new insights into the mechanisms of chronic renal failure and help to identify new targets for arresting and/or reversing the development of the disease.

## Introduction

Several studies estimate that the number of patients affected by CKD is in sharp rise in western countries [1]. Decline of renal function can be slowed down by therapies targeting the renin angiotensin system (RAS) but they are partially effective [2]. Therefore, dialysis or transplantation are the only available options allowing the survival for the majority of patients whom number requiring such treatment is steadily increasing worldwide. Thus, proposing more effective treatments for arresting or even better reversing the progression of CKD is one of the major challenges of public health today.

CKD can be promoted by a variety of mechanisms including hypertension which may affect any of the kidney structures by promoting development of chronic inflammation leading to fibrosis and progressive decline of renal function [3]. One of the main objectives of our research group over the last years has been the identifications of molecular mechanisms responsible for the development of renal fibrosis. For this purpose, we use a multi-target strategy based on experimental models of nephropathies in rodents to study mediators of inflammation, progression, stabilization or regression of renal lesions [4]–[8]. Even if these experimental models had provided new insights towards the progression and regression of CKD, a relative limitation would have been the fact that renal disease was induced in young animals for a rather short period of time. Consequently in these models, animals did not suffer for a long period of time from a chronic disease such as hypertension or diabetes as it is usually the case in humans. Thus, it can be argued that the mechanisms of progression were very fast, diverging from the slow progression of CKD in humans and inversely that the demonstrated efficiency of therapy was mainly due to the young age of animals.

For this purpose we used the RenTg mouse, a novel hypertensive transgenic model that overexpresses renin at high and constant levels after genetic clamping hypothesizing that it will progress with a slower rate towards CKD. In a recently published first study, we addressed the issue of whether aged mice, suffering for a long period from hypertension, can be efficiently treated using RAS antagonists [9]. Indeed, we found that the decline of renal function, as well as alterations in the expression of proteins involved in the integrity and the function of the kidney, were partially reversible when 13 month old animals were treated by an AT1 receptor antagonist [9]. In the present study, we investigated the kinetics and the physiopathological events at the cellular and molecular level that delineate the pathogenic process leading to progressive development of renal disease associated to the activation of RAS. We propose that the RenTg strain is a useful model to study the different phases of progression and to identify the pathological pathways involved in the evolution of CKD.

## Results

### RenTg mice Develop Hypertension-induced Progressive Renal Disease

**Figure 1:**
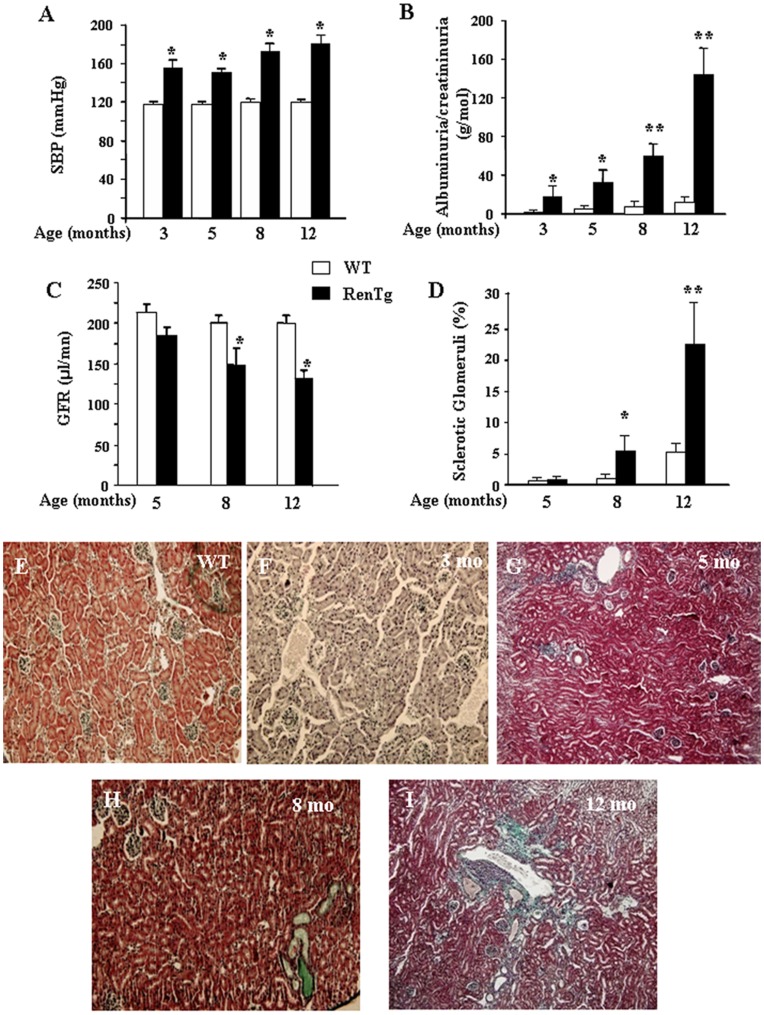
RenTg mice are hypertensive and develop chronic kidney disease progressively with the age. Systolic blood pressure was highly increased in RenTg mice since the age of 3 months as compared to aged matched WT controls (A). Urinary excretion of albumin progressively increased with age to reach at 12 months highly elevated values in RenTg mice (B) whereas GFR significantly decreased (C). In accordance, the percentage of sclerotic glomeruli progressively increased in RenTg mice compared to WT mice (D). Representative examples of renal cortical slices after Masson’s Trichrome histology (E–I). Renal cortex of WT (E) as well as 3 month-old RenTg appeared normal (F); inflammatory lesions and renal fibrosis were evident at 5 (G), and 8 (H) months respectively in RenTg mice. Renal cortical tissue of 12 month old RenTg mice displayed several of the characteristics of hypertension-associated renal disease such as peri- vascular and glomerular inflammation, glomerulosclerosis, fibrinoid-like deposits within renal vessels, glomerular ischemia and tubular dilatation (I). Values are means ± SEM; n = 8 and 9 for wild type and RenTg mice, respectively for each time point; *P<0,05 or **P<0,01 vs WT. Magnification of the microphotographs: X200.

RenTg mice were generated as already described [10]. We have recently shown that these mice display high blood pressure associated to an advanced phase of renal disease (12 month old mice). To study sequential events leading to progressive CKD we measured arterial blood pressure and examined alterations of kidney structure and function in RenTg mice and littermates from 3 to 11 month old animals. As expected, RenTg mice displayed elevated systolic blood pressure from 3 month old (153±7 mmHg compared to 120±2 for the WT age matched animals, p<0.05, Fig. 1A). Proteinuria was slightly augmented at 3 months and progressively increased with age to reach at 11 months highly elevated values as compared to the WT control animals (5.15±0.09 g/mol and 130±20 g/mol of creatinine for WT and RenTg mice respectively, p<0.01, Fig. 1B). Decline of renal function was also confirmed by glomerular filtration rate (GFR) measurements and glomerulosclerosis evaluation. RenTg mice showed a progressive decrease of GFR (Fig. 1C). In accordance, the percentage of sclerotic glomeruli was significantly increased at 8 months in RenTg mice (Fig. 1D). As illustrated by Masson’s trichrome, at the early stages of the disease (3 month old) renal morphology appeared to be normal (Fig. 1F) whereas moderate infiltrate was detected in 5 month-old RenTg mice (Fig. 1G). Histological examination at 8 months showed marked tubular dilatation (Fig. 1H). Finally, kidneys of 12 month-old RenTg displayed well-established lesions in all renal compartments such as peri- vascular and glomerular inflammation, glomerulosclerosis, fibrinoid-like deposits within renal vessels, glomerular ischemia and tubular dilatation (Fig. 1I).

### Altered Expression of Cell Adhesion Markers in RenTg mice

**Figure 2:**
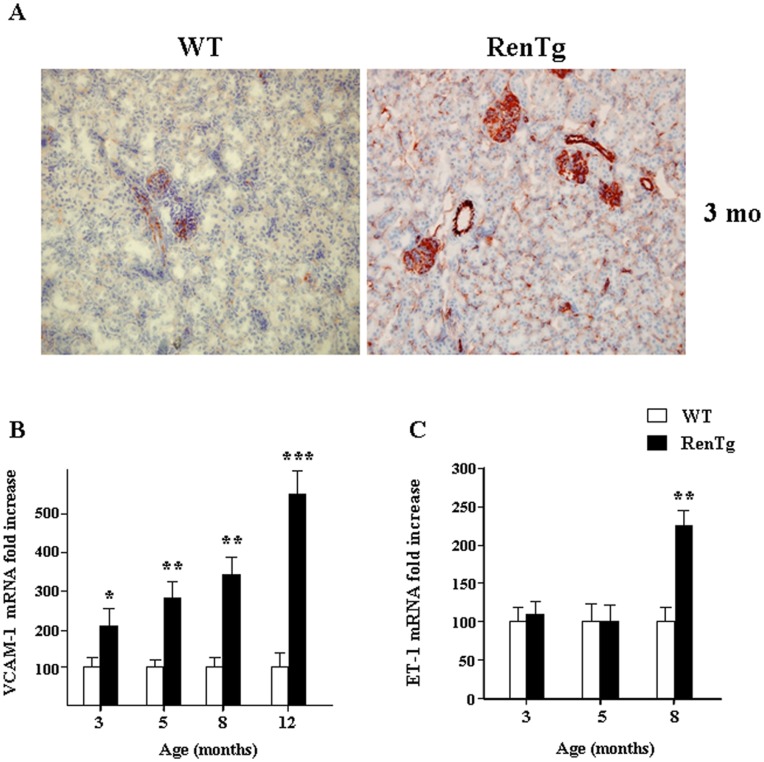
Alterations of endothelial function markers in RenTg mice. Immunohistochemistry on renal cortical cryosections showed a marked increase of the PECAM-1 (CD31) expression in the 3 month old RenTg mice as compared to the control tissues (A). Quantitative RT-PCR analysis showed a progressive increase of VCAM-1 in the RenTg mice (B). Note that the ET-1 mRNA expression is up-regulated at the later stages of the renal disease (C). Values are presented as fold increase as compared to the age matched WT mice and are means ± SEM; n = 8 and 9 for wild type and RenTg mice, respectively for each time point; *P<0,05 or **P<0,01 vs WT. Magnification of the microphotographs: X200.

Endothelial dysfunction is considered as the starting point of injury in various inflammatory diseases and can be initiated in part by alterations of cell adhesion markers (CAMs) expression. Immunostaining in the renal cortex of 3 month-old RenTg mice revealed a pronounced upregulation of the endothelial marker CD31/PECAM-1 (platelet endothelial cell adhesion molecule-1) in the peritubular microcirculation as well as in the glomerular and vascular endothelium (Fig. 2A). In addition, mRNA levels for vascular adhesion molecule-1 (VCAM-1) which is essential for the recruitment of inflammatory cells were continuously increased from 3 months (Fig. 2B) as well as those of the intercellular cell adhesion molecule-1 (ICAM-1, data not shown). mRNA for endothelin-1 (ET-1) was upregulated only during the later time points of the disease (Fig. 2C). These data showed that the expression of several CAMs was altered at the early stages of the disease, indicating that endothelial dysfunction may play a leading role in the development of CKD.

### Inflammatory Cell Infiltration in RenTg mice

**Figure 3:**
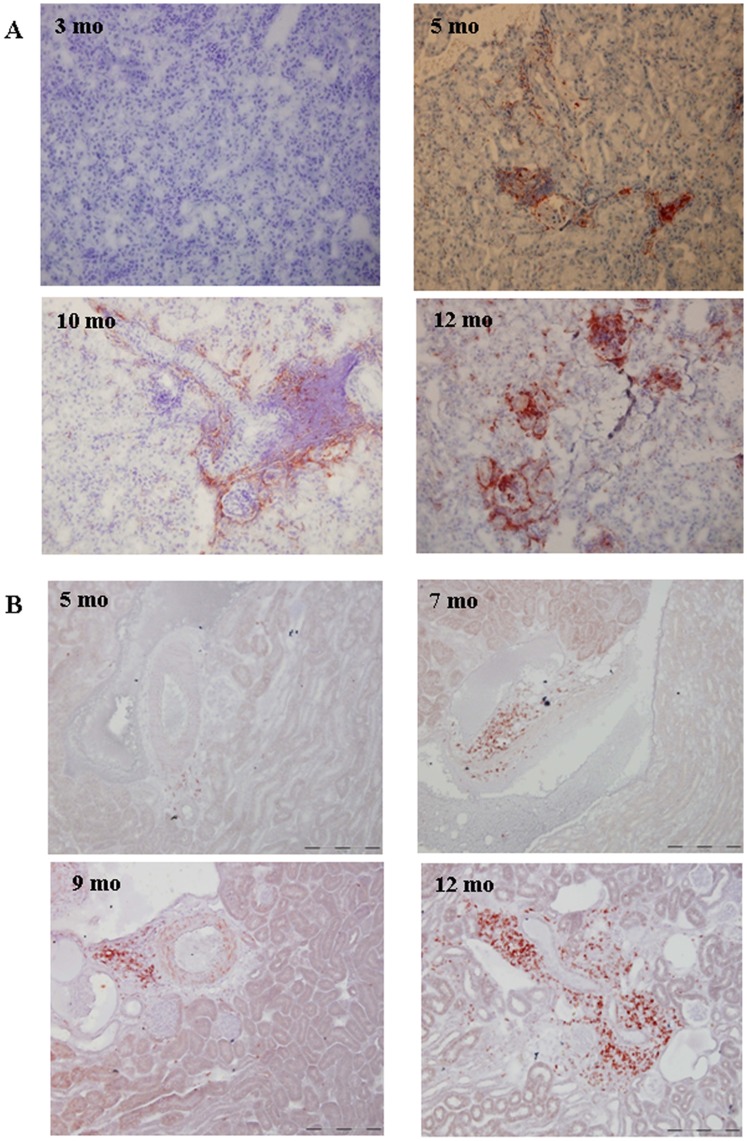
Inflammatory cell infiltration during the progression of CKD in RenTg mice. Representative examples of renal cortical sections for macrophages (CD68) and lymphocytes (CD3) immunostaining, respectively. Note that macrophage infiltration was first detected since the age of 5 months in the renal cortex of RenTg mice (A), whereas a positive staining for lymphocytes was observed at 7 month-old RenTg mice (B). Magnification of the microphotographs: X200.

Upregulation of CAMs initiates adhesion of inflammatory cells in the damaged tissues. Thus, the next step was to study inflammation in the injured kidneys of RenTg mice during the progression of chronic kidney disease. Immunostaining for CD68 revealed some macrophage infiltration in cortical slides of the 5 month-old in RenTg mice (Fig. 3A). The infiltrate was much more significant at the latter stages of the disease. In addition, lymphocyte infiltration was detected at a later age (7 months) and became more pronounced in 12 month-old RenTg mice as indicated by CD3 immunostaining (Fig. 3B). CD68 and CD3 immunostainings were negligible for the WT animals at each time point (data not shown). In conclusion, the inflammatory process appeared after alteration of CAMs expression during progression of CKD in RenTg mice.

### Progression of Renal Fibrosis in RenTg mice

**Figure 4:**
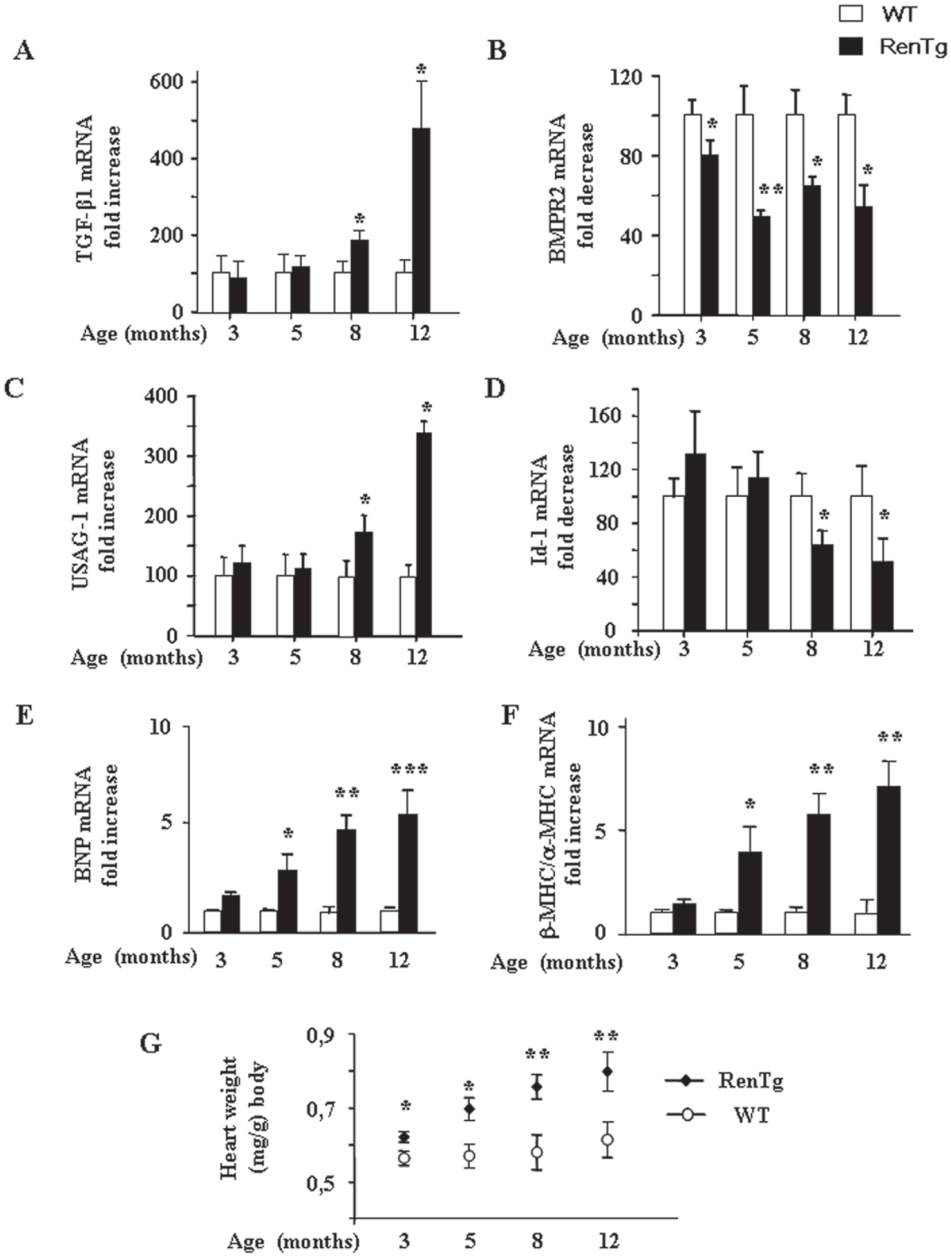
Changes in the TGFβ/BMP balance and cardiac hypertrophy during the progression of CKD in RenTg mice. Quantitative RT-PCR analysis of TGF-β1 (A), BMPR2 (B), USAG-1 (C) and Id-1 (D) in the renal cortex of wild type and RenTg mice. Note that the expression of BMPR2 has been decreased since 3 months in RenTg mice while at the expressions of TGF-β1 and USAG-1, an endogenous inhibitor of BMPs, were highly increased from 8 months in injured tissues. Significant down regulation of Id-1 mRNA expression, a BMP7 target gene is also detected after 8 months. In addition, qPCR analysis of typical markers of cardiac hypertrophy, such as BNP (E) and β-MHC/α-MHC ratio (F), as well as measurements of the heart weight (H), confirmed that RenTg mice developed cardiac hypertrophy with age. Values are presented as fold increase or decrease as compared to the age matched WT mice and are mean ± SEM; n = 8 and 9 for wild type and RenTg mice, respectively for each time point; *P<0,05 or **P<0,01 vs WT.

Matrix remodelling commonly follows inflammatory response and is characterized by changes in the TGFβ/BMP balance. Consequently, we measured gene expressions of pro- and anti-fibrotic genes of the TGFβ/BMP superfamily at different phases of CKD progression. As shown in Figure 4A mRNA expression for TGFβ1 was upregulated since 8 months and peaked high levels at 12 months. The renal expression of several members of the BMPs family (BMP-2, BMP-4, BMP-6, BMP-7) which are known to antagonize TGFβ actions did not change throughout the development of CKD in all tested ages (data not shown). However, the expression of BMPR2, a BMP receptor, was down regulated from the early stages of the disease (Fig. 4B). Interestingly, the expression of agents acting as endogenous inhibitors of BMPs, such as uterine sensitization-associated gene-1 (USAG-1), was markedly upregulated in the renal cortex of the RenTg mice at the latest stages of renal disease (Fig. 4C), whereas Id-1 mRNA expression, a BMP-pathway target gene, was several folds decreased (Fig. 4D). To examine whether overexpression of renin could compromise cardiac function we also measured some typical markers of cardiac hypertrophy such as brain natriuretic peptide (BNP) and the ratio of β/α myosine heavy chain (MHC) (Fig. 4E and 4F respectively). As expected, mRNA expression of these two markers was highly increased indicating that RenTg mice develop cardiac hypertrophy with age. Similar results were observed by estimating the heart weight reported to the body weight of the animals (Fig. 4G).

**Figure 5:**
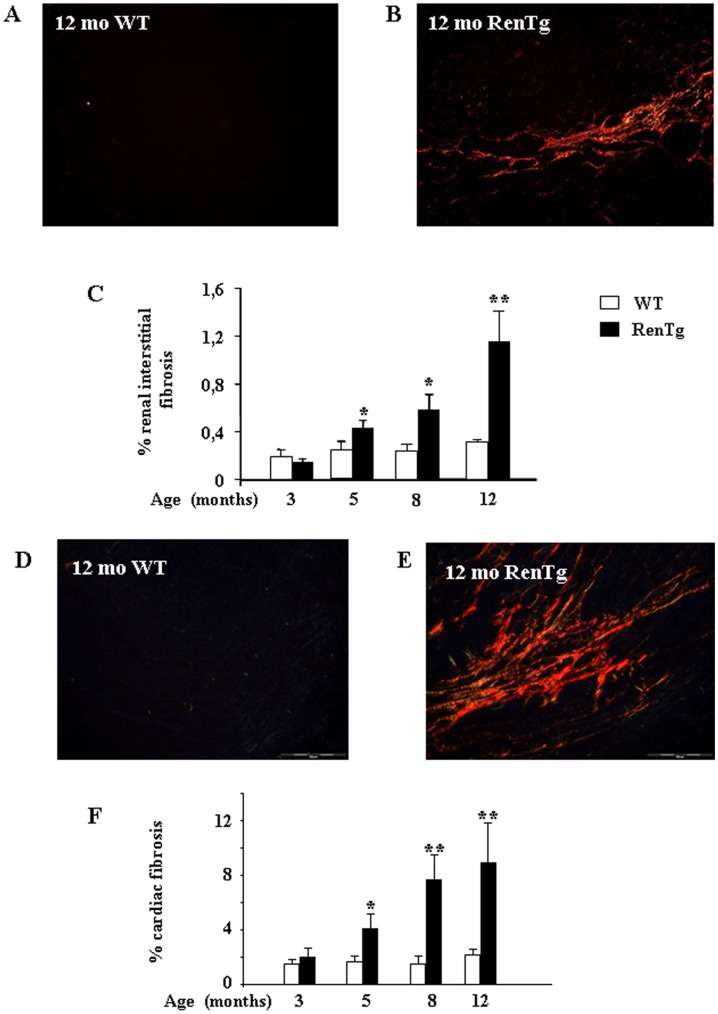
Progression of renal interstitial and cardiac fibrosis in hypertension-induced CKD. Representative example of Sirius red staining in WT (A) and RenTg (B) 12 month-old mice. Quantification by morphometric analysis of renal cortical paraffin embedded sections after Sirius Red staining is shown in panel C. Fibrillar collagen content was progressively increased in RenTg mice after 5 months. Similar results were observed in heart sections of WT (D) and RentTg (E) 12 month-old mice. Quantification of cardiac fibrosis is shown in panel F. Values are presented as fold increase as compared to the age matched WT mice and are means ± SEM; n = 8 and 9 for wild type and RenTg mice, respectively for each time point; *P<0,05 vs WT.

In accordance with real-time PCR experiments, Sirius red staining showed that collagen deposition was progressively increased in the renal cortex (Fig. 5A, 5B and 5C) as well as in cardiac tissues (Fig. 5D, 5E and 5F) of injured animals from the age of 5 months. These results indicate that the equilibrium of TGFβ/BMP balance was shifted towards the pro-fibrotic agents of this family after the initial endothelial and inflammatory events.

### Altered Expression of Proteins Characterizing the Integrity and/or Phenotype of Podocytes and Proximal Tubular Cells

**Figure 6:**
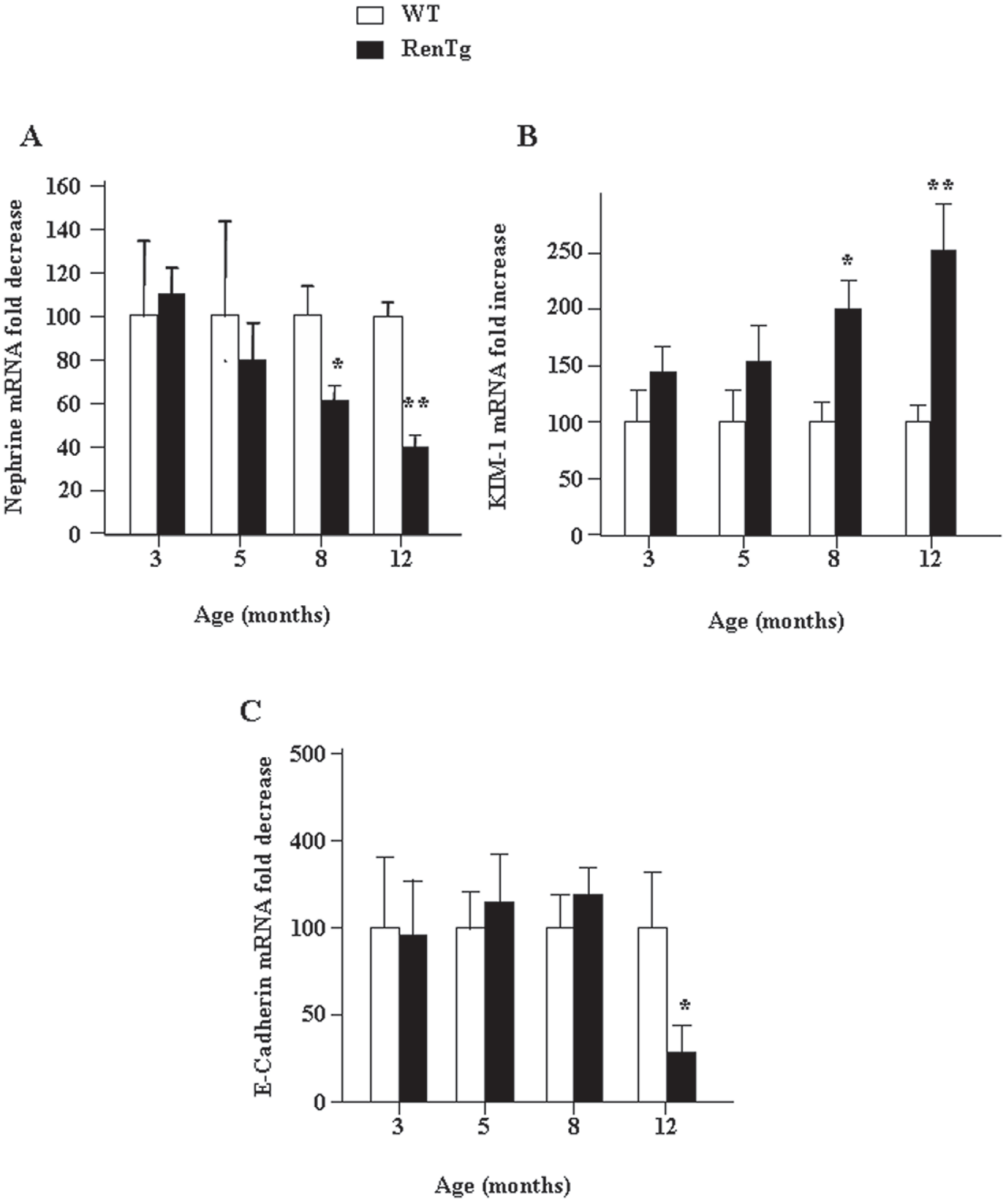
Alterations of mRNA expression of podocyte and tubular markers during progression of CKD in RenTg mice. Quantitative RT-PCR analysis of nephrin (A), KIM-1 (B) and E-cadherin (C) in the renal cortex of wild type and RenTg mice. Note that the expression of nephrin is decreased since 8 months in RenTg mice while at the same age the expression of KIM-1 was highly increased. Significant differences in E-cadherin expression were detected in the 12 month old RenTg mice. Values are presented as fold increased or decreased as compared to the age matched WT mice and are mean±SEM; n = 8 and 9 for wild type and RenTg mice, respectively for each time point; *P<0,05 or **P<0,01 vs WT.

To provide cellular assessment of the previous described modifications we studied the expression of two markers essential for the normal structure and function of the kidney. Figure 6A shows that expression of the nephrin mRNA, a protein essential for the normal structure of podocytes, started to decrease in RenTg mice relatively late (after 8 months). Similarly, the expression of kidney injury molecule-1 (KIM-1), an indicator of renal tubular damage, was two-fold upregulated at 8 months compared to their respective control animals (Fig. 6B). Interestingly, E-cadherin, a tubular marker of normal epithelial phenotype was decreased only at the latest stages of the renal disease (Fig. 6C). These data suggest that the phenotypic alterations in podocytes occur during the intermediate to advanced stages of renal disease and are associated to tubular stress signals while the loss of the proximal tubule phenotype is a rather late event in this model of CKD.

In conclusion, our data indicate that the development of CKD in RenTg mice results from a continuous decline of renal function associated with sequential events such as endothelial dysfunction, inflammation and alterations in the TGFβ/BMP equilibrium, all leading to structural changes within all renal compartments.

## Discussion

During the last years, the incidence of CKD is in sharp rise in western countries, mainly because of population ageing, improved survival from cardiovascular diseases and epidemic spreading of type-2 diabetes. Thus, the prevention, arrest and/reversal of decline of renal function are major public health priorities as current treatments are only partially effective. A better understanding of the mechanisms involved in this pathology is essential to develop more effective and specific therapies. CKD may be caused by diseases that affect any of the renal structures, including vessels, glomeruli and the tubule-interstitial compartment. The damage can be induced by various pathologies; the most prominent are hypertension and diabetes [11]–[12]. These processes are linked by their ability to promote inflammatory reactions and tissue scaring that destroys the structure and the function of individual nephrons.

Persistent RAS activation plays a major role in renal disease progression [13]. Indeed, regression of proteinuria and renal fibrosis by blocking the RAS action has been reported in some experimental models of CKD in rodents, such as chronic nitric oxide deficiency, infusion of AngII by osmotic minipumps, nephrectomy or puromycin aminonucleoside [6], [14]–[16]. However, a potential limitation of these studies is that the disease was induced in young animals not suffering for a long period from a chronic disease like hypertension or diabetes. Thus, all these models of kidney injury are very fast with kinetics deviating a lot from the development of CKD. Another complication in these animal models is the uncontrolled homeostatic feedback that occurs subsequent to genetic or experimental manipulations. In addition, in some cases therapy was induced before reaching high levels of proteinuria and a significant level of renal structural damage. To address these issues we used a novel model of Ang II-induced renal disease which we hypothesized could mimic closer the kinetics and the physiopathological characteristics of hypertension-associated renal disease. This strain, also known as RenTgKC mice, is viable, fertile and suitable thus for studying sequential events leading to progressive development of renal disease. In addition, by expressing the transgene in the liver, driven by a liver-specific promoter/enhancer, the ectopically produced active renin is completely freed from normal renal homeostatic adjustments, and is therefore clamped. Furthermore, because the transgene is of mouse origin, the resulting high circulating active renin can interacts with other components of the mouse RAS leading to a severe Ang II-mediated hypertension and to kidney damage [10]. Of note, we preferred to use the RenTgKC model instead of the RenTgMK one, another variant of the RenTg mice, because of the lower expression of the renin transgene RenTgKC mice don’t die rapidly from heart failure and they can live long enough to develop renal disease.

In models of hypertension-induced renal disease, such as nitric oxide deficiency or infusion of AngII by osmotic minipumps, there is a progressive increase of the blood pressure during the first 3–5 days. Thereafter blood pressure is stabilized at the same high level for weeks. In contrast, renal structure is not altered during the first weeks. Depending on the species and the genetic background the first visible alterations appear after few or several weeks and the renal structure continues to deteriorate with time, although blood pressure is unchanged. Rentg mice mimic to a slower, thus more progressive, rate the above-mentioned models. Blood pressure is increasing gradually during the first weeks of life, and then is stabilized and then the structural and functional alterations appear and continue to be deteriorated with time. Similar observations are also described in spontaneously hypertensive rats.

Among the first major cellular events observed in RenTg mice was a significant increase of the CAM expression. It is generally believed that the vascular endothelium serves as a barrier by providing a non adherent surface to inflammatory cells. However, upon Ang II stimulation, endothelium becomes dysfunctional as the expression of CAMs and chemokines, such as VCAM-1, ICAM-1 and MCP1, is upregulated thus promoting infiltration of inflammatory cells within the damaged tissue [17]–[19]. Therefore, an impairment of the resident endothelium may contribute to the initiation of chronic renal injury. In addition, an upregulation of the ET-1 production, which is absent in the normal kidney, was observed at the later stages of the disease in RenTg animals. We have previously demonstrated that ET-1 is an important mediator of the fibrogenic action of Ang II which can activates its synthesis [20]. Of note, at 8 months RenTg mice presented increased expression of ET-1 which corroborated with installation of interstitial fibrosis.

Changes in the TGFβ/BMP balance control the regulation and relative expression between pro and anti-fibrotic members and can play a crucial role in the progression of CKD. It has been recently shown that exogenous administration of BMP7 reversed renal fibrosis in some animal models of CKD [21]–[22]. Although we didn’t observe a significant decrease in its endogenous levels, we found an early decrease of the expression of BMP receptors. As the disease progressed, TGFβ and its cofactors were upregulated together with a parallel increase in the expressions of endogenous inhibitors of BMPs. These altered expressions in the pro-fibrotic members of the TGFβ superfamily were associated to the development of fibrotic lesions. These results are in agreement with previous observations showing that overexpression of USAG-1 accompanied renal fibrosis, whereas blockade of AT1 receptor and reversal of renal disease was associated with the inhibition of USAG-1 activation [9], [23]–[24]. It appears thus, that the development of renal fibrosis is a complex phenomenon involving several mechanisms such as a direct fibrogenesis through activation of TGFβ accompanied with an inhibition of BMPs action either by down-regulation of the BMP receptor and/or activation of BMPs endogenous antagonists.

Proteinuria is a major marker of the severity of renal disease and predicts the risk of progression [12]. Elevated proteinuria is basically associated with the disruption of slit diaphragm and loss of podocyte foot processes, structural alterations that used to be considered as irreversible. We have previously shown ultrastructural modifications in the glomerular structure of 12 months old RenTg mice [9]. In particular, podocyte foot processes disappeared while glomerular basement membranes displayed abnormal thickness. In the present study we found that the alterations in the expressions of proteins indicating phenotype changes in podocytes and tubular epithelium (such as nephrin, E-cadherin, KIM-1) are rather late events and occur well after the appearance of the inflammatory and fibrotic lesions.

In conclusion, our data indicate that the development of CKD in RenTg mice results from a continuous decline of renal function associated with sequential events such as endothelial dysfunction, inflammation and alterations in the TGFβ/BMP equilibrium, all leading to structural changes within all renal compartments. We can therefore assume that RenTg mice can be cross-bred with other transgenic strains to provide double transgenic animals suffering from progressive hypertension and CKD, lacking or over-expressing a given protein to study its implication in the disease. Consequently, such a model may constitute a powerful tool to study new insights on the mechanisms involved in the progression and regression of chronic renal disease.

## Materials and Methods

### Animals

Experiments were performed using RenTg mice backcrossed in the genetic background 129SV as already described [9]. These mice express renin ectopically at a constant high level in the liver leading to elevated mRNA (2.7±0.3 renin/18S versus 0 in RenTg and WT mice respectively) and protein levels of active renin into the blood stream [10]. Thus, RenTg mice are hypertensive as endogenous synthesis of angiotenin II is increased. Male RenTg and WT counterparts were sacrificed at 3, 5, 8 and 12 months. At least 8 mice per group, and for each time point, have been used for the experiments. Kidneys were removed, decapsulated, and the cortex was dissected from the medulla. The cortical tissue is then used for RNA extraction and immunohistochemistry.

All mice were kept in well-controlled animal housing facilities and had free access to tap water and pellet food. All protocols were performed according to the French Institutional Committee guidelines (INSERM and the University of Pierre et Marie Curie, Paris).

### Measurement of Systolic Arterial Pressure

Systolic arterial pressure (mmHg) was measured using a tail-cuff sphygmomanometer adapted to the mouse as described [9]. Briefly, animals were accustomed for three weeks before measurements. Only not stressed mice that showed stable and reproducible values of blood pressure for at least three consecutive days were considered for the measurements. Ten measurements from each mouse were taken at two minutes intervals then a mean value was determined. To avoid variations due to day cycle all blood pressure measurements were carried out between 9 and 11a.m.

### Measurements of Urinary Albumin Excretion

RenTg mice and aged-matched controls were transferred in metabolic cages with free access to food and water for 24-hour urine collection. Measurements of microalbuminuria were performed as previously reported [9]. Urinary albumin concentration was normalized to urinary creatinine concentration, and values were expressed as g albumin/mol creatinine.

### Glomerular Filtration Rate

GFR was measured using the single bolus injection method of FITC-inulin as previously described [25]. Briefly, RenTg mice and their littermates (n = 5 mice per group) were injected retro-orbitally under isoflurane anesthesia with 3.74 µl/g of body weight 5% FITC-inulin solution (Sigma-Aldrich). Blood samples were collected every 15 minutes during 90 minutes and plasma was isolated after centrifugation. GFR was estimated and expresses in µl/min ± SEM.

### Renal Morphology and Evaluation of Interstitial Fibrosis

One kidney from each animal was fixed in formalin solution (4%), embedded in paraffin after conventional processing, and sections (3 mm thick) were stained with Masson’s trichrome for histological evaluation, glomerulosclerosis and Sirius red for analysis of fibrillar collagen. Interstitial fibrosis was assessed semi-quantitatively on Sirius red stained paraffin sections at magnification of X200. Fibrosis was then quantified using computer-based morphometric analysis software (Analysis, Olympus) that allowed the formation of a binary image in which the stained area could be automatically calculated as percentage of the image area. To obtain a percentage of glomeruloscelrosis, the number of sclerotic glomeruli were referred to the totality of glomeruli evaluated for each mouse (mean = 90 glom/mouse). Ten fields per section that covered nearly the whole piece of cortex were randomly selected. Scoring was performed in blind on coded slides.

### Immunohistochemistry

Immunostainings were performed on frozen fixed with acetone for 3 min and paraffin embedded 3 mm thick sections, placed onto super Frost*H*glass slides (Menzel GmbH & Co KG). Paraffin sections were deparaffinized and rehydrated with baths of xylene and graded alcohol. Antigens were unmasked with citrate buffer (pH = 6) and proteinase K. Sections were treated with peroxidase for 10 min in order to block endogenous peroxidase activity followed by 10 min incubation with avidin and biotin. Between each incubation period, sections were washed in PBS. The antibodies used were anti-CD3 (1/100, Santa Cruz), anti-CD68 (1/100, (Serotec, Raleigh, NC) and anti-CD31 (1/100, Abcam, MA). All steps were performed at room temperature. These samples were analyzed using an Olympus BX50 inverted microscope. All immunostainings have been confirmed using at least 5 mice from each group. Negative controls included omission of primary antibodies or preincubation of primary antibodies with immunogenic peptides.

### Total RNA Extraction and Quantitative Real Time PCR

Total RNA was extracted from renal cortex using TRIzol reagent (Invitrogen) according to the manufacturer’s instructions. RNA quality was checked by control of optical density at 260/280 nm. Contaminating genomic DNA was removed by RNase-free DNAse (Qiagen) for 15 min at room temperature. cDNA was synthesized from 1 µg of purified RNA using oligo-dT and superscript II RT (Qiagen) for 1 h30 at 37°C and 10 min at 70°C. Real-time PCR amplification was performed with Roche Light Cycler 480 Sequence Detection System using SYBR Green PCR Master Mix (Qiagen) as previously described [Huby AC 2009]. All samples were assayed in triplicate, and the average value of the triplicate was used for quantification. Results are expressed as the ratio of a given gene/gene reference (HPRT or 18S) cDNA. All primers used for the study are listed in Table 1.

**Table 1:** List of the primers used for the Real Time-PCR of the different genes as mentioned in the results sections.

Gene		Primers
Nephrin	sense	AGCTCTGCATCCAAGAAGCAGTA
	antisense	AGTTGTCTCTGAGGTGCCTTTGA
E-cadherin	sense	GTGCCGGCTTCACTTTCA
	antisense	GGAGTAGGCTTGGACCTTGTC
KIM1	sense	CCCACGCTACCTCTGCTC
	antisense	GATGGATACCTGAGCATCACC
ET-1	sense	GTTGGCTCAGCCAGATGCA
	antisense	AGCCTACTCATTGGGATCATCTTG
TGF-β1	sense	TCTGGCAGTGTGGACTGG
	antisense	CAAGGAAGCTTGGGGACAT
VCAM1	sense	TGGTGAAATGGAATCTGAACC
	antisense	CCCAGATGGTGGTTTCCTT
HPRT	sense	TCCTCCTCAGACCGCTTTT
	antisense	CCTGGTTCATCGCTAATC
18S	sense	GAGCGAAAGCATTTGCCAAG
	antisense	GGCATCGTTTATGGTCGGAA
BMPR2	sense	GAGCCCTCCCTTGACCTG
	antisense	GTATCGACCCCGTCCAATC
USAG-1	sense	GCAACAGCACCCTGAATGAAG
	antisense	TGTATTTGGTGGACCGCAGTT
Id-1	sense	CCAGTGGGTAGAGGGTTTGA
	antisense	AGAAATCCGAGAAGCACGAA

### Statistical Analyses

Values are expressed as mean±SEM. All data were analyzed using one-way ANOVA followed by Protected Least Significant Difference Fisher’s test of the Statview software package. Results with P<0.05 were considered statistically significant.
